# Genetic diversity modulates the physical and transcriptomic response of skeletal muscle to simulated microgravity in male mice

**DOI:** 10.1038/s41526-023-00334-8

**Published:** 2023-12-01

**Authors:** Yasmina Zeineddine, Michael A. Friedman, Evan G. Buettmann, Lovell B. Abraham, Gabriel A. Hoppock, Henry J. Donahue

**Affiliations:** Department of Biomedical Engineering, Virginia Commonwealth University, Richmond, VA USA

**Keywords:** Genetics research, Translational research

## Abstract

Developments in long-term space exploration necessitate advancements in countermeasures against microgravity-induced skeletal muscle loss. Astronaut data shows considerable variation in muscle loss in response to microgravity. Previous experiments suggest that genetic background influences the skeletal muscle response to unloading, but no in-depth analysis of genetic expression has been performed. Here, we placed eight, male, inbred founder strains of the diversity outbred mice (129S1/SvImJ, A/J, C57BL/6J, CAST/EiJ, NOD/ShiLtJ, NZO/HILtJ, PWK/PhJ, and WSB/EiJ) in simulated microgravity (SM) via hindlimb unloading for three weeks. Body weight, muscle morphology, muscle strength, protein synthesis marker expression, and RNA expression were collected. A/J and CAST/EiJ mice were most susceptible to SM-induced muscle loss, whereas NOD/ShiLtJ mice were the most protected. In response to SM, A/J and CAST/EiJ mice experienced reductions in body weight, muscle mass, muscle volume, and muscle cross-sectional area. A/J mice had the highest number of differentially expressed genes (68) and associated gene ontologies (328). Downregulation of immunological gene ontologies and genes encoding anabolic immune factors suggest that immune dysregulation contributes to the response of A/J mice to SM. Several muscle properties showed significant interactions between SM and mouse strain and a high degree of heritability. These data imply that genetic background plays a role in the degree of muscle loss in SM and that more individualized programs should be developed for astronauts to protect their skeletal muscles against microgravity on long-term missions.

## Introduction

As the field of space exploration progresses towards missions to Mars, the development of permanent lunar bases, and the privatization of space travel, there is a concomitant need to accelerate our understanding of how to protect astronauts against the dangers of long-term missions. A primary risk of spaceflight is skeletal muscle deconditioning, which stems from the microgravity-induced unloading of the musculoskeletal system^1,2^. Apart from the increased risk of injury associated with losing skeletal muscle mass and strength^3^, reloading weakened skeletal muscle fibers after exposure to microgravity can lead to injury^4–7^. The need to transition between environments of varying gravitational field strengths without experiencing impaired locomotor function or injury emphasizes the importance of conserving skeletal muscle during exposure to microgravity^8^. In simulated microgravity (SM), skeletal muscle mass appears to reach steady state at 70% of the original size after 270 days of unloading^1^, and the fastest trip to Mars with current technology will take at least 220 days^9^, suggesting a high risk for significant muscle loss during future missions. Current nutrition and exercise interventions have been shown to attenuate microgravity-induced skeletal muscle atrophy; however, long-term missions still lead to significant reductions in muscle skeletal mass and strength^10–15^, prompting the need for additional countermeasure strategies against muscle loss.

What is interesting about the skeletal muscle response to unloading is the range seen in the degree of muscle atrophy between individuals. Significant variability is observed between astronauts in terms of their skeletal muscle responses to spaceflight and the inter-subject response to bed rest^12,16–19^. Recorded volumetric losses from baseline in the skeletal muscles of the legs range between 6 and 24% in spaceflight and bed rest studies^20–24^. Similarly, losses in knee extensor and plantar flexor strength have ranged from 0 to 29% for missions between 11 and 84 days^20,25,26^ and 0–55% for missions between 30-380 days, respectively^11,27,28^. Within this variability, one consistent result is that the soleus, gastrocnemius, and quadriceps, known as anti-gravity muscles, are the most susceptible to unloading-induced atrophy. Their role in bipedal motion and maintenance of upright posture means that their atrophy leads to impairments in locomotion and balance upon return to stronger gravitational fields^7,12,21,29^. Determining the underlying causes of variability can enhance the development of both generic and personalized countermeasures to unloading-induced skeletal muscle atrophy, especially that of the anti-gravity muscles.

Several factors partially explain the variability in skeletal muscle response to microgravity, including preflight fitness levels and adherence to nutritional and exercise guidance during missions^30–33^. However, the genetic component has been largely ignored. Muscle mass and strength have been shown to have high levels of heritability (phenotypic variance explained by genotype) in several studies with estimates of heritability of muscle mass ranging from 50 to 80% and muscle strength from 30 to 85%^34^. Considering the significant genetic influence on baseline muscle mass and strength, it is reasonable to assume that genetics also severely affects how muscles respond to unloading. Judex et al. found a quantitative trait loci on chromosome 5 that was responsible for 5% of the variability in muscle cross-sectional area loss in response to unloading in mice^35^. Maroni et al. investigated the role of genetic variability in the skeletal muscle response to disuse in founder strains of diversity outbred (DO) mice. Founder DO mice consist of eight inbred strains of mice, chosen to maximize genetic diversity and that have an extensively sequenced genome. When crossed, they form the DO mouse population, which makes for ideal models for genetics-based studies^36^. Maroni et al. placed five founder DO strains in single-hindlimb immobilization for three weeks. A mouse strain-dependent effect on disuse-induced changes in muscle mass and protein synthesis was found, suggesting that genetics play a role in the skeletal muscle response to disuse^37^. However, no studies to date have identified specific differentially expressed genes across individuals of different genetic backgrounds that could be responsible for regulating individual responses to microgravity.

In this study, we placed the eight founder strains of DO mice into hindlimb unloading (HLU), a well-studied model of microgravity, and investigated the role of genetics in both the transcriptomic and physical response of skeletal muscles to SM induced by HLU. Previous studies in our lab using these DO founder mice demonstrated that genetic variability affects the response of bone to SM^38^. We collected data on lower limb skeletal muscle morphology, twitch force, protein synthesis markers, and RNA expression. Our hypothesis was that the morphological, biological, and transcriptomic responses of the eight founder DO strains to SM would be mouse strain-dependent.

## Results

### Genetic variation affected the magnitude of changes in body weight and skeletal muscle mass

**Fig. 1:**
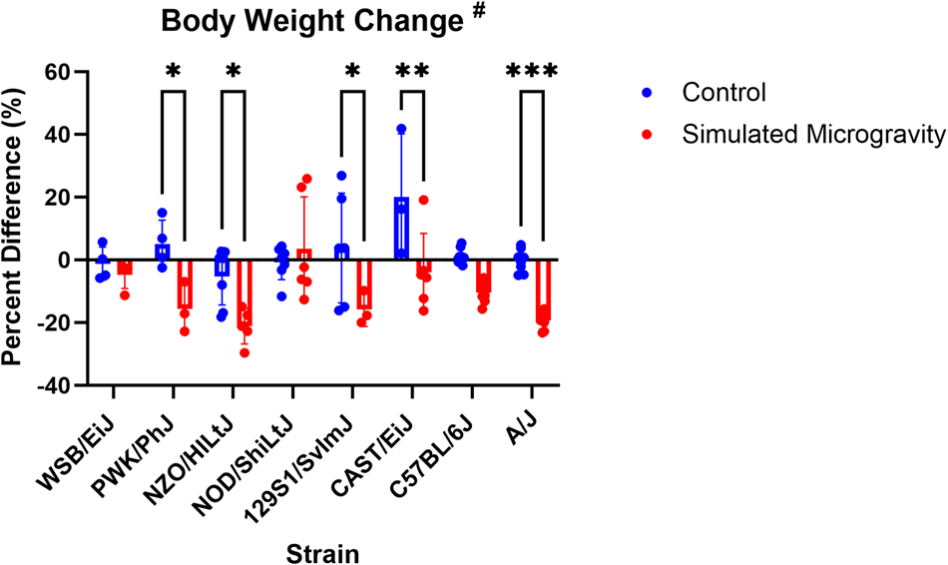
Body weight change. Change in body weight (mean ± SD) from day 1 to day 23 presented as a percent difference from day 1. A/J mice demonstrated the most significant difference between the SM and control groups (*p* < 0.001). NZO mice in SM experienced the greatest magnitude of weight loss. Analysis was performed with two-way ANOVA with Tukey’s Multiple Comparisons test (*n* = 3–8). # represents a significant interaction between strain and unloading with *p* < 0.05. (*p < 0.05; **p < 0.01; ***p < 0.001; ***p < 0.0001; simulated microgravity vs control within the same strain).

BW was analyzed on days 1 and 23 of SM (Fig. 1). There was a significant (*p* = 0.0051) interaction between mouse strain and SM changes in BW. This interaction indicates that SM caused weight loss in some mouse strains while other mouse strains were protected from this effect. Five of the eight strains (PWK/PhJ, NZO/HILtJ, 129S1/SvImJ, CAST/EiJ, A/J) had significantly greater weight loss in the SM groups than in their control groups. Raw body weight data can be found in Supplementary Data File 1.

**Fig. 2:**
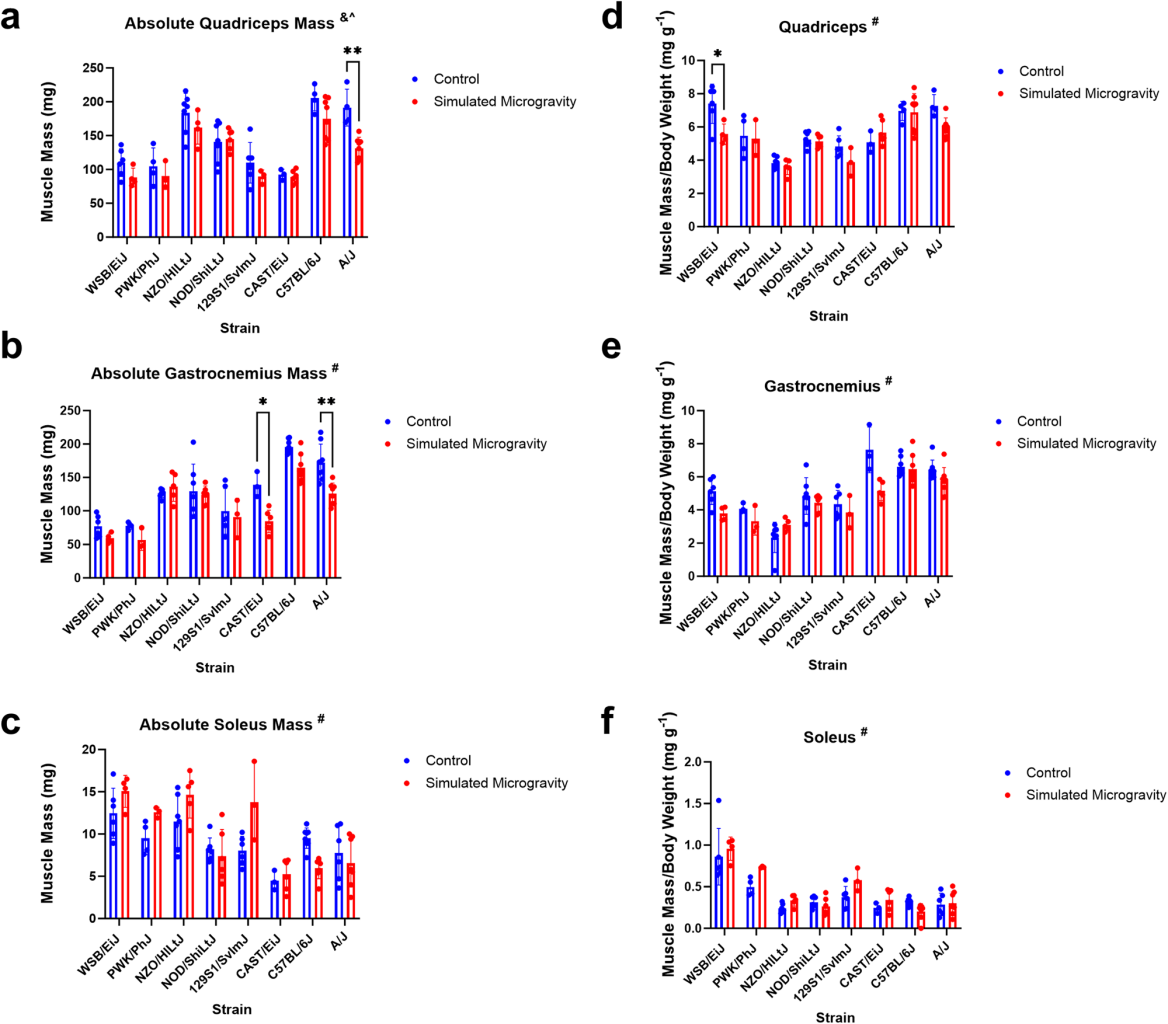
Muscle mass. **a**–**c** Absolute wet mass in mg (mean ± SD) of the quadriceps, gastrocnemius, and soleus after three weeks in control or simulated microgravity. **d**–**f** Muscle mass of the quadriceps, gastrocnemius, and soleus normalized to body weight (mg/g) after three weeks of control or simulated microgravity conditions. A/J mice had significantly smaller quadriceps and gastrocnemius absolute masses after exposure to simulated microgravity. CAST/EiJ mice had smaller gastrocnemius absolute masses after exposure to simulated microgravity. Analysis was performed with two-way ANOVA (*n* = 3–8). & represents a main effect of strain; ^ represents a main effect of unloading; # represents a significant interaction between strain and unloading (*p* < 0.05). (*p < 0.05; **p < 0.01; simulated microgravity vs control within the same strain).

Skeletal muscle masses of quadriceps and gastrocnemius were analyzed in terms of absolute mass and mass normalized by BW (Fig. 2). Raw muscle mass data can be found in Supplementary Data File 1. Baseline variation in muscle masses between strains necessitates normalizing muscle mass to BW. A significant interaction between mouse strain and SM was found for all absolute and normalized masses (quadriceps/BW: *p* = 0.0232; gastrocnemius: *p* = 0.015; gastrocnemius/BW: *p* = 0.0035; soleus: *p* = 0.0005; soleus/BW: *p* = 0.0412), except for absolute quadriceps, which had a significant strain-dependent and unloading-dependent effect but not interaction. A/J mice demonstrated significantly lower absolute quadriceps in SM than control (*p* = 0.0064), but this effect disappears when normalized to BW (*p* = 0.29) as seen in Fig. 2a and b. WSB/EiJ mice demonstrated significantly lower quadriceps mass/BW in SM than control (*p* = 0.0297). Both A/J and CAST/EiJ SM mice had significantly lower absolute gastrocnemius masses than their respective controls (*p* = 0.0046; *p* = 0.0488). These effects also disappear when masses are normalized to BW. There were no significant differences between SM and control for gastrocnemius mass/BW, absolute soleus mass, and soleus mass/BW for all mouse strains.

### Cross-sectional muscle area and volume in response to SM varied by mouse strain

**Fig. 3:**
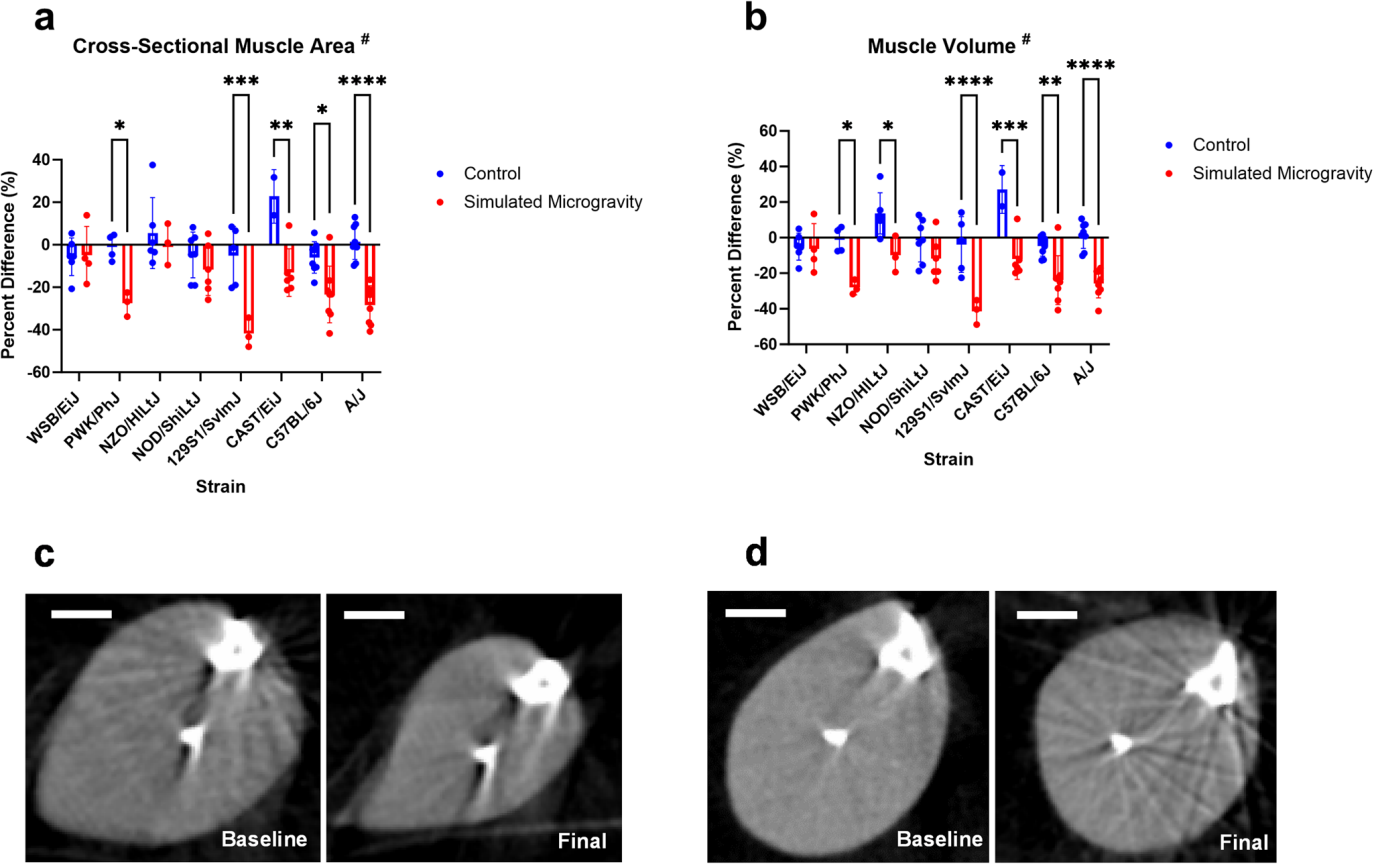
Changes in muscle cross sectional area and volume. **a** Changes in lower leg cross-sectional skeletal muscle area represented as percent difference between day 1 and day 23. **b** Changes in lower leg skeletal muscle volume represented as percent difference from day 1 to day 23. **c** Baseline (left) and final day (right) mid-diaphyseal cross-sectional area slice from an A/J mouse. **d** Baseline (left) and final day (right) mid-diaphyseal cross-sectional area slice from an NOD/ShiLtJ mouse., depicted as mean ± SD. The lower leg is defined as the segment between where the tibia and femur interface to the distal end of the tibia. A/J mice had the most significant differences in muscle volume and cross-sectional area in SM compared to control. 129S1/SvImJ mice in SM experienced the largest magnitude of loss in cross-sectional area and volume in SM compared to control. Analysis was performed with two-way ANOVA with Tukey’s post hoc test (*n* = 3–8). # represents a significant interaction between strain and unloading (*p* < 0.05). (*p < 0.05; **p < 0.01; ***p < 0.001; ****p < 0.0001; simulated microgravity vs control within the same strain). The scale bars represent 1.5 mm.

Cross-sectional muscle area and volume were analyzed using microCT scans of the lower limbs of the mice (Fig. 3). A significant interaction between mouse strain and SM was found for both skeletal muscle cross-sectional area and volume (*p* = 0.0009; *p* = 0.0042). PWK/PhJ, 129S1/SvImJ, CAST/EiJ, C57BL/6J, and A/J mice exposed to SM all experienced significant losses in lower limb cross-sectional muscle area after three weeks. These same strains as well as NZO/HILtJ all experienced significant reductions in lower leg muscle volume after three weeks of SM.

### Muscle strength

**Fig. 4:**
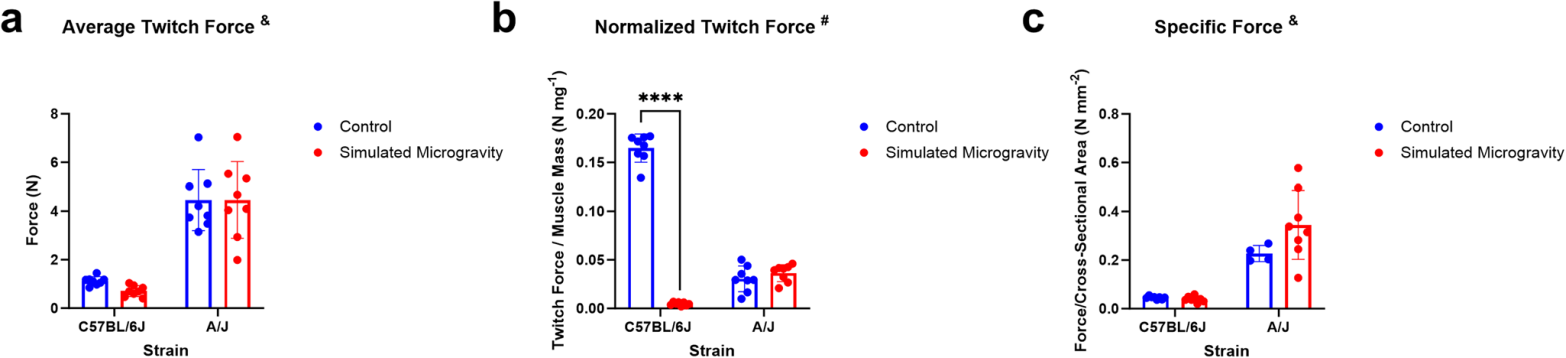
Muscle strength. **a** Average absolute twitch force in C57BL/6J and A/J mice. **b** Twitch force normalized to gastrocnemius mass. **c** Twitch force normalized to average cross-sectional area, also known as specific force. All forces were measured from plantar flexion induced by peripheral nerve stimulation, and data is shown as mean ± SD. Figure 5a, c shows baseline differences in twitch force and specific force between the C57BL/6J and A/J strains but no significant differences in response to SM within strains. Figure 5b shows a significant loss in normalized twitch force in C57BL/6J mice in response to simulated microgravity. Analysis was performed with two-way ANOVA test (*n* = 3–8). & represents a main effect of strain; # represents a significant interaction between strain and unloading (*p* < 0.05). (****p < 0.0001 simulated microgravity vs control within the same strain).

Muscle strength was analyzed using plantar flexion force measurements. Twitch force was normalized to skeletal muscle mass of the gastrocnemius to account for variation in muscle mass between strains. A significant interaction between mouse strain and SM was found for normalized twitch force (*p* < 0.0001). For average twitch force, only mouse strain was found to be a significant source of variation (*p* < 0.0001). C57BL/6J mice had significantly weaker (*p* < 0.0001) absolute twitch forces than A/J mice in control groups (Fig. 4b). When the twitch force was normalized to skeletal muscle mass, only C57BL/6J mice displayed significantly lower forces after three weeks of SM (*p* < 0.0001).

### RNA sequencing revealed genetic variation affected the transcriptomic response to SM

Across the eight strains there were a total of 84 DEGs and 935 differentially regulated gene ontologies (GOs, FDR < 0.05) in response to SM (Table 1). Gene expression varied between strains following three weeks of SM. By three weeks, A/J mice had the strongest response to SM with 68 DEGs and 328 differentially regulated GOs, followed by CAST/EiJ mice, who had 11 DEGs and 99 differentially regulated GOs. NOD/ShiLtJ mice had the fewest number of DEGs (0) and differentially regulated GOs (5) in response to SM. Three genes were universally affected by SM: *Nus1, Chrnb1*, and *Dusp8* (Table 2). The most affected GO pathways in response to SM were related to muscle contraction, protein transcription and translation, and metabolic processes (Table 3). Expanded RNA sequencing datasets can be found in Supplementary Data File 2.

**Table 1:** Number of differentially expressed genes, gene ontologies, and enrichment pathways by strain.

Strain	Upregulated DEGs	Downregulated DEGs	Upregulated gene ontologies	Downregulated gene ontologies	Upregulated enrichment pathways	Downregulated enrichment pathways
All strains	1	2	8	51	0	54
WSB/EiJ	0	0	0	32	0	17
PWK/PhJ	1	0	59	20	84	12
NZO/HILtJ	0	0	51	77	43	60
NOD/ShiLtJ	0	0	0	5	0	1
129S1/SvImJ	0	0	0	163	0	49
CAST/EiJ	8	3	69	30	16	32
C57BL/6J	1	0	32	10	16	32
A/J	57	11	30	298	36	163

**Table 2:** Expression and significance levels of selected differentially expressed genes by strain.

Mouse strain	Gene symbol	Gene name	logFC	AveExpr	*P*-value	FDR
All strains	Dusp8	Dual-specificity phosphatase 8	−0.67	4.72	4.23E−08	0.000557
	Nus1	Nus1 dehydrodolichyl diphosphate synthase subunit	−0.32	4.79	1.30E−06	0.008634
	Chrnb1	Cholinergic receptor, nicotinic, beta polypeptide 1	0.73	5.61	8.10E−07	0.005331
A/J	Junb	Jun-B transcription factor	−3.02	6.39	9.12E−07	0.005852
	Serpine1	Plasminogen activator inhibitor 1 precursor	−2.03	4.53	2.98E−06	0.012752
	Cdkn1a	Cyclin dependent kinase inhibitor 1 A	−1.45	6.84	4.73E−06	0.015166
	Slc41a3	Solute carrier family 31-member 3	−1.07	5.19	6.52E−06	0.016732
	Thbs1	Thrombospondin 1	−1.91	4.66	8.39E−06	0.017946
	Slc19a2	Solute carrier family 19-member 2	−2.16	2.81	1.15E−05	0.018566
	Ppid	Peptidylprolyl isomerase d	−1.03	4.43	1.16E−05	0.018566
	Nfkbiz	NF-kappa-B inhibitor zeta	−2.19	2.02	1.52E−05	0.021733
	Tnfaip6	TNF alpha induced protein 6	−1.72	1.61	1.96E−05	0.025145
	Ccl7	C-C motif chemokine ligand 7	−5.81	1.09	2.98E−05	0.028223
	Gfpt2	Glutamine-fructose-6-phosphate transaminase 2	−2.01	4.15	3.27E−05	0.028223
	Cxcl1	C-X-C motif chemokine ligand 1	−5.53	1.35	3.85E−05	0.028223
	Actg1	Actin, gamma, cytoplasmic 1	−0.86	7.88	8.39E−05	0.037107
	Piezo1	Piezo-type mechanosensitive ion channel component 1	−1.23	5.56	8.91E−05	0.038105
	Krt18	Keratin 18	2.64	2.73	9.93E−05	0.041079
	Clu	Clusterin	−1.11	4.96	1.07E−04	0.041911
	Ednrb	Endothelin receptor type B	−1.77	4.17	1.08E−04	0.041911
	Il1r2	Interleukin 1 receptor, type II	−2.86	0.59	1.16E−04	0.043065
	Cish	Cytokine inducible SH2-containing protein	−2.32	5.03	1.18E−04	0.043065
	Ncam1	Neural cell adhesion molecule 1	1.44	3.49	1.21E−04	0.043065
	Socs3	Suppressor of cytokine signaling 3	−4.55	5.06	1.46E−04	0.044308
	Adm	Adrenomedullin	−1.33	2.99	1.47E−04	0.044308
	Egr1	Early growth response 1	−2.48	6.77	1.48E−04	0.044308
	Coq10b	Coenzyme Q-binding protein COQ10 homolog B, mitochondrial	−0.87	4.52	1.49E−04	0.044308
	St8sia2	ST* alpha-N-acetyl-neuraminide alpha-2,8-sialyltransferase 2	4.12	−1.26	1.62E−04	0.044308
	Icam1	Intercellular adhesion molecule 1	−1.74	3.52	1.83E−04	0.045353
	Cxcl2	Chemokine C-X-C motif ligan 2	−5.12	−1.12	2.01E−04	0.045949
	Ccl2	Chemokine C-C motif ligand 2	−5.49	1.41	2.36E−04	0.047988
CAST/EiJ	Cpz	Carboxypeptidase Z	−5.05	−1.01	6.24E−06	0.034057
	Myoz2	Myozenin 2	1.68	7.73	1.37E−05	0.034057
	Atp2a2	Sarco(endo)plasmic reticulum calcium-ATPase 2 (SERCA2)	1.58	8.68	1.38E−05	0.034057
	Ampd3	Adenosine monophosphate deaminase 3	1.58	2.64	1.74E−05	0.034057
	Ankrd2	Akyrin repeat domain 2	2.78	6.66	1.87E−05	0.034057
	Prkag3	Protein kinase, AMP activated, gamma 3 non-catalytic subunit	−1.26	6.89	1.90E−05	0.034057
	Tnnc1	Troponin C1	1.61	7.68	2.22E−05	0.034057
	Lmod2	Leiomodin 2	1.98	6.58	2.61E−05	0.034057
	P2ry6	Pyrimidinergic receptor p2y6	−1.89	1.84	2.87E−05	0.034057
	Synj2	Synaptojanin 2	1.38	4.91	3.06E−05	0.034057
C57BL/6J	Sh2d3c	Sh2 domain containing 3c	0.79	3.59	0.000002	0.021532
PWK/PhJ	S100a9	S100 calcium binding protein a9 (calgranulin B)	6	−2.02	0.000002	0.025252

**Table 3:** Gene ontology analysis by strain in simulated microgravity compared to control.

Strain	Upregulated gene ontology biological process	*P*-value	FDR	Downregulated gene ontology biological process	*P*-value	FDR
All	DNA Metabolic Process	4.6e−05	0.048	Negative regulation of angiogenesis	7.9e−06	0.012
	Sarcomere organization	0.0001	0.049	Response to unfolded protein	9.7e−06	0.012
	Striated muscle contraction	0.00013	0.049	Telomere maintenance via telomerase	0.00011	0.027
WSB/EiJ				Response to unfolded protein	6e−09	1.3e−05
				‘de novo’ posttranslational protein folding	5.2e−07	0.00056
				Chaperone mediated protein folding requirement cofactor	1.4e−06	0.001
PWK/PhJ	Cotranslational protein targeting to membrane	3.2e−32	7.3e−29	Actin-myosin filament sliding	3.e−07	0.00029
	Protein targeting to ER	4.3e−30	3.2e−27	Striated muscle contraction	2.5e−06	0.0013
	rRNA metabolic process	6.1e−18	1.3e−15	Glycogen catabolic process	3.4e−06	0.0013
NZO/HILtJ	Striated muscle contraction	1.9e−11	2.8e−08	Negative regulation of cellular process	2.5e−08	6.3e−05
	Actin-myosin filament sliding	1.4e−09	5.2e−07	Extracellular matrix organization	1.4e−06	0.0017
	Sarcomere organization	4.4e−08	1.4e−05	Regulation of angiogenesis	1e−05	0.0062
NOD/ShiLtJ				Negative regulation of transcription, DNA-templated	1.7e−05	0.02
				Myelination	3.1e−05	0.024
				Negative regulation of transcription from RNA polymerase II promoter	9.6e−05	0.046
129S1/SvImJ				Positive regulation of cell differentiation	1.2e−10	3.1e−07
				Cellular amino acid catabolic process	2.1e−09	2.8e−06
				Regulation of cell migration	1.5e−06	0.0005
CAST/EiJ	Muscle contraction	3e−11	4.8e−08	Extracellular matrix organization	4.4e−12	8.9e−09
	Actin-myosin filament sliding	7.8e−11	4.8−08	Peptide metabolic process	3.2e−07	0.00027
	Myofibril assembly	8.3e−11	4.8e−08	Collagen fibril organization	3.9e−07	0.00027
C57BL/6J	Regulation of transcription from RNA polymerase II promoter	5.5e−10	1.3e−06	Respiratory chain complex IV assembly	2.8e−05	0.022
	RNA 3’ end processing	2.5e−06	0.0018	mRNA processing	3.2e−05	0.022
	Termination of RNA polymerase II transcription	4.7e−05	0.016	Polyamine biosynthetic process	0.00029	0.074
A/J	SRP-dependent Cotranslational protein targeting to membrane	5.8e−13	1.1e−09	Cellular response to cytokine stimulus	6.1e−24	1e−20
	Peptide biosynthetic process	8e−08	2.2e−05	Cytokine-mediated signaling pathway	7.4e24	1e−20
	Translation	1.7e−06	0.00032	Positive regulation of transcription from RNA polymerase II promoter	3.5e−12	2.5e−09

### p-p70S6K1 increased in response to SM in WSB mice

**Fig. 5:**
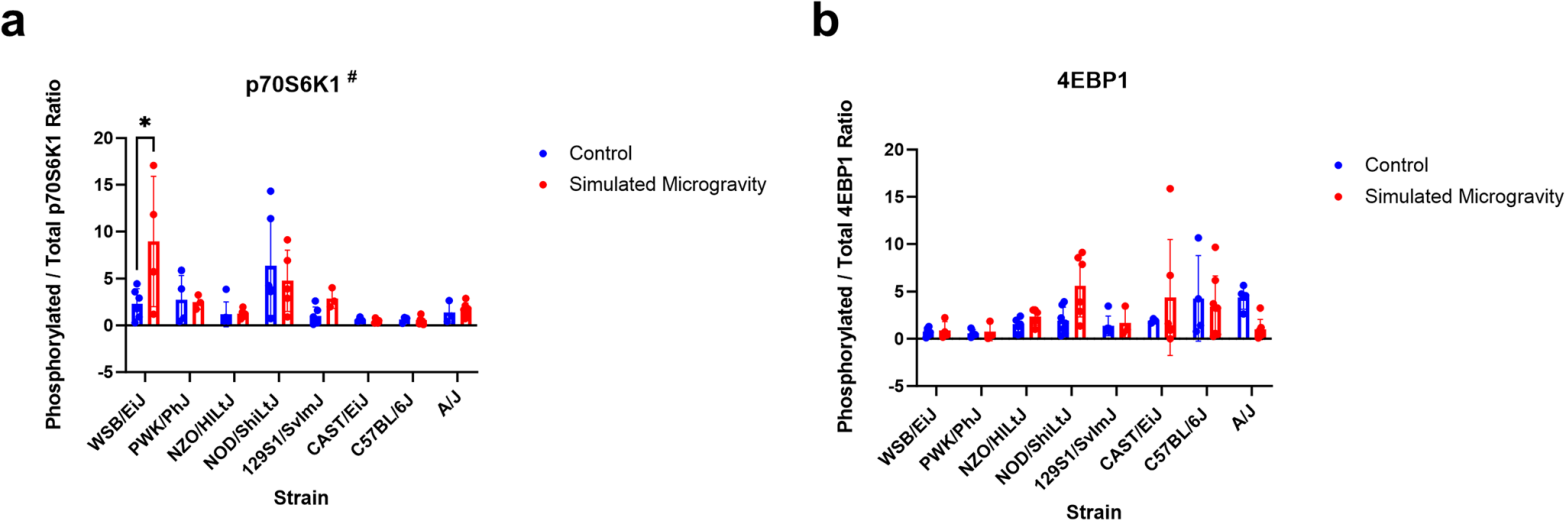
Protein synthesis markers. **a** The ratio between phosphorylated and total p70S6K1 expression (mean ± SD) in the left quadriceps of each strain after three weeks in control or simulated microgravity conditions. **b** The ratio between phosphorylated and total 4EBP1 expression (mean ± SD) in the left quadriceps of each strain after three weeks in control or simulated microgravity. All blots were derived from the same experiment and processed in parallel. Analysis was performed with two-way ANOVA (*n* = 3–8). # represents a significant interaction between strain and unloading (*p* < 0.05). (*p < 0.05 simulated microgravity vs control within the same strain).

p70S6K1 and 4EBP1 are markers of protein synthesis within the mTOR pathway. The phosphorylation of these proteins was measured in the left quadriceps. There was a significant interaction between mouse strain and SM in p-p70S6K1/total p70S6K1 expression (*p* = 0.002) (Fig. 5a). Only WSB/EiJ mice had significantly higher (*p* = 0.0153) p-p70S6K1/total p70S6K1 after three weeks in the SM group compared to the control group (Fig. 5a). No other strains showed differences in p-p70S6K1/total p70S6K1 between control and SM. No significant differences were found between SM and control mice in terms of p-4EBP1/total 4EBP1 expression.

### Heritability

Narrow-sense heritability, a measure of the additive effects of genetics on phenotypic variance, was calculated for several properties (Table 4). The percentages reflect the variance in the response to SM due to genetic factors. Several properties showed high levels of heritability in response to SM, including soleus/BW at 83.2%, gastrocnemius mass at 82.5%, and quadriceps mass at 78.7%. Changes in muscle area and muscle volume after SM had heritability values of 55.5% and 48.3%, respectively. In control conditions, body weight has the highest heritability at 93.8%, followed by gastrocnemius and quadriceps mass normalized to body weight. Most properties have a higher level of heritability in response to SM compared to control. Soleus muscle mass and soleus/BW heritability are much higher in response to SM (74.2%, 83.2%) than control (49.3%, 67.3%). Heritability of phosphorylation of 4EBP1 in response to SM is 19.9% compared to 42.2% in control. Together, the data shows a high degree of heritability in the skeletal muscle response to SM.

**Table 4:** Heritability analysis of skeletal muscle properties.

Property	Control	Simulated microgravity
BW	93.8%	96.2%
Change in BW	31.2%	52.7%
Muscle Mass–Quadriceps	73.5%	78.7%
Muscle Mass–Gastrocnemius	77.3%	82.5%
Muscle Mass–Soleus	49.3%	74.2%
Muscle Mass/BW–Quadriceps	77.6%	68.6%
Muscle Mass/BW–Gastrocnemius	80.2%	77.1%
Muscle Mass/BW–Soleus	67.3%	83.2%
p-4EBP1	42.2%	19.9%
p-p70S6K1	38.5%	60.0%
Change in muscle area	30.7%	55.5%
Change in muscle volume	44.3%	48.3%
Baseline muscle area	61.8%	50.1%
Baseline muscle volume	68.6%	62.5%

## Discussion

This study investigated how genetic variation affects the response to three weeks of SM in the eight founder strains of DO male mice using muscle morphology and RNA sequencing data. The results suggest that genetics significantly influence the skeletal muscle response to SM, supported by variation in loss of BW, muscle size, gene expression, and muscle function by strain. A/J mice demonstrated the greatest sensitivity to SM as evidenced by the most significant differences in BW, muscle morphology, and gene expression compared to other strains. In comparison, NOD/ShiLtJ mice were most protected and had the least sensitive response to SM and demonstrated little to no responses to SM in terms of muscle morphology, protein expression, or gene expression.

BW and muscle morphology data show that there was a significant interaction between mouse strain and skeletal muscle response to SM (Figs. 1–3). BW was reduced in SM in NZO/HILtJ, PWK/PhJ, A/J, 129S1/SvImJ, and CAST/EiJ mice with NZO/HILtJ mice losing up to 30% BW. Food intake was not measured in this study; however, several investigations have demonstrated significant losses in both BW and muscle mass occur after unloading independent of changes in food intake^37,39–42^. A/J mice were the most susceptible strain to SM-induced muscle loss based on skeletal muscle mass and volume changes. There were no significant effects of SM on soleus muscle mass (Fig. 2). C57BL/6 J mice appeared to have a reduction of about 30% in soleus muscle mass in SM, but this difference was not statistically significant (Fig. 2c, *p* = 0.2641). Previous studies demonstrate a 21-45% loss in soleus mass after 3–5 weeks of unloading^43–45^. The soleus muscle is known to be highly susceptible to unloading-induced atrophy, so the lack of significant differences between SM and control in any strain was an unexpected result. However, the lower limb skeletal muscle response has only been extensively characterized in C57BL/6J mice of the eight strains in this study, meaning the other strains could have protective mechanisms that delay or attenuate the loss of soleus muscle following exposure to SM. Losses in muscle volume and cross-sectional area were seen in PWK/PhJ, NZO/HILtJ 129S1/SvImJ, CAST/EiJ, C57BL/6J, A/J mice and PWK/PhJ, 129S1/SvImJ, CAST/EiJ, C57BL/6J, A/J, respectively (Fig. 3). These data show that muscle mass and morphology as well as BW responses to SM are affected by genetics.

Decreases in postnatal muscle size during SM are typically due to atrophy of individual muscle fibers rather than decreases in the number of muscle fibers, a process mediated by imbalances in protein synthesis and degradation^46,47^. The degree of muscle atrophy in response to disuse has been shown to vary significantly in humans between individuals^12,26,29^. The primary pathway responsible for the maintenance of skeletal muscle is mTORC1, in which mechanical load, IGF-1, or Akt act as anabolic factors that trigger downstream signaling to activate p70S6K1 and 4E-BP1 and lead to protein synthesis. Inhibition of mTORC1 leads to upregulation of MAFbx (Muscle Atrophy F-box)/Atrogin-1 and MuRF1 (Muscle Ring Finger-1), which promote protein degradation^48^. Interestingly, despite A/J and CAST/EiJ mice demonstrating considerable losses in skeletal muscle, western blots revealed no reductions in the phosphorylation of protein synthesis markers p70S6K1 or 4EBP1 in the quadriceps. While in humans, unloading leads primarily to reduced protein synthesis, rodents have been shown to respond through early, transient reductions in protein synthesis (<1 week following unloading), followed by increased protein degradation^49–51^. This could explain why protein synthesis appeared unaffected after three weeks. It is more likely that by this stage, protein degradation would be the primary mechanism by which muscle loss occurs. In the pathway enrichment analysis, the mTOR pathway was indeed found to be downregulated in SM compared to control for all strains (FDR = 0.056). WSB/EiJ mice, who showed lower quadriceps/BW in SM compared to control, had upregulated p-p70S6K1/total p70S6K1 in SM compared to control (Fig. 4). Qualitative observations of WSB/EiJ mice in SM showed that they were more active than other mice and constantly tried to find leverage on their food pellets or water bottles for standing, which could explain the increased expression of p-p70S6K1.

A/J mice were the most sensitive to SM, as evidenced by the greater losses in skeletal muscle mass, volume, and body weight compared to other mice (Figs. 1–3), in addition to the greater number of DEGs and differentially expressed GOs (Tables 1–2). A/J mice were bred to be a model for cancer and tend to develop tumors following exposure to carcinogens. They have also been reported to have weaker responses to pathogens and are susceptible to the spontaneous development of autoimmune diseases^52,53^. These patterns suggest that the A/J strain has a dysregulated immune response compared to other strains. Of note within the 68 DEGs in the A/J mice was the downregulation of *Piezo1, Cxcl2, Ccl2*, and *Ccl7. Piezo1* encodes a mechanosensitive ion channel known to mediate cellular responses to loading. *Piezo1* expression decreases during disuse and plays a role in the upregulation of atrophy-related genes during muscle immobilization^54^. The downregulation of *Piezo1* seen in the most SM-sensitive strain supports the association between disuse atrophy and *Piezo1* inhibition. *Cxcl2, Ccl2*, and *Ccl7* are chemokines with anabolic effects on muscle tissue^55–60^. Their decreased expression in SM, combined with the downregulation of cytokine-mediated signaling pathway and cellular response to cytokine stimulus GOs, suggest there was suppression or alteration of immune function occurring in A/J mice exposed to SM that contributed to muscle loss. Spaceflight is known to induce immune dysregulation^61,62^ and the immune system also plays a role in mediating the skeletal muscle response to disuse and recovery^63–66^. The baseline immune dysfunction in A/J mice could have been exacerbated by SM and led to the particularly sensitive response of A/J to SM. This finding suggests that immune dysfunction experienced during spaceflight could increase the magnitude of skeletal muscle loss and that enhancing the strength of the immune system could attenuate skeletal muscle loss during spaceflight, though further investigation is needed to confirm this link.

Three genes were universally differentially expressed (*p* < 0.05) in SM compared to control groups: *Dusp8* (FC of −0.67), *Chrnb1* (FC: 0.73), and *Nus1* (FC: −0.32). These three genes could be used as targets for countermeasures against unloading-induced skeletal muscle atrophy. *Dusp8* dephosphorylates mitogen-activated protein kinases, including p38, JNK, and ERK1/2. Expression of ERK1/2 is associated with the conversion from fast to slow twitch muscle fibers^67,68^, and its inhibition induces upregulation of Atrogin-1 and MuRF1^69^. MuRF1 is regulated by p38 activation in HLU^70^. Downregulation of *Dusp8* implies reduced inhibition of ERK1/2 and p38, suggesting muscle fiber type remodeling and muscle atrophy pathways were active in all SM mouse strains. *Chrnb1* encodes the beta subunit of the skeletal muscle acetylcholine receptor. Acetylcholine subunit upregulation is seen in several unloading models^71–77^, and is indicative of neuromuscular dysfunction. However, the beta subunit positively regulates receptor turnover and metabolic half-life when phosphorylated^78^. It is possible that its upregulation could simultaneously be a countermeasure against neuromuscular junction instability in response to SM. *Nus1* encodes the neurite outgrowth inhibitor B receptor (NgBR). NgBR is involved in several biomolecular processes, notably cholesterol trafficking^79^, angiogenesis^80^, neural development^81^, and dolichol synthesis, which is required for an important and ubiquitous post-translational protein modification known as protein-N-glycosylation^82^. Downregulation of *Nus1* could imply alterations in the rate of protein processing, decreased angiogenesis, or buildup of intracellular cholesterol deposits, processes previously reported in skeletal muscle undergoing SM^83–87^. Together, these three DEGs show a universal response to SM irrespective of genetic background consisting of neuromuscular instability, skeletal muscle remodeling, and alterations in protein processing pathways.

GO analyses revealed further variation in transcriptomic response to SM between mouse strains (Table 2). PWK/PhJ was the only strain that demonstrated downregulations in muscle-specific pathways, such as striated muscle contraction and actin-myosin filament sliding. NZO/HiLtJ and CAST/EiJ mice, on the other hand, showed upregulations in muscle contraction, actin-myosin filament sliding, and sarcomere organization, which could be protective mechanisms meant to counteract any SM-induced muscle loss. Transcription and translation-related pathways were both upregulated and downregulated across most of the strains, indicating some level of remodeling is occurring within the skeletal muscles. PWK/PhJ, C57BL/6 J, and WSB/EiJ strains had downregulated metabolic pathways, including respiratory chain complex assembly and glycogen metabolism, which could be a result of reduced energy production due to decreased muscle usage. Across all strains, telomere maintenance via telomerase was downregulated, suggesting that the preservation of telomeres is affected by SM regardless of genetics. Telomeres tend to be shorter after long-duration spaceflight^88,89^ a pattern associated with age-related pathologies in humans^90^ and hematopoietic deficiencies in mice^91^. The GO analysis shows strain-dependent transcriptional responses to SM and a universal effect of SM on telomere regulation.

C57BL/6J mice showed significant losses of strength (*p* < 0.0001) following three weeks of SM compared to those in the control group, while A/J mice showed no significant differences (Fig. 5). C57BL/6J mice showed a decrease in strength without significant changes in muscle mass. Decreases in strength are shown to precede, and often exceed, muscle atrophy during unloading, thought to be a result of neuromuscular junction degeneration, decreased neural drive to the muscles, and altered patterns of motor unit recruitment^27,92–94^. RNA sequencing revealed that A/J mice in SM had upregulations in *Ncam1* (FC: 1.44) and *St8sia2* (FC: 4.12)*. Ncam1* and *St8sia2* are thought to encourage neural tissue regeneration following damage by promoting neuron outgrowth and differentiation^95–99^. These transcriptomic responses in A/J mice suggest active neuromuscular junction remodeling in the skeletal muscles of the A/J mice and potentially predict decreases in muscle strength. C57BL/6J mice showed no significant differential regulation of neuromuscular genes or pathways, suggesting that these changes may have occurred earlier in the experiment and stabilized by week three. These data show differences in the timeline of how muscle strength responds to SM between strains. Future studies should have DEG analyses from several timepoints throughout the experiment to better illustrate strain-based differences in the response to SM over time.

Heritability analyses support and expand on previous results. For instance, we observed that change in BW and muscle mass in response to SM are highly heritable^37^. Heritability of quadriceps/BW and gastrocnemius/BW in response to SM were 69% and 77% respectively, compared to 70% and 51% in response to single-hindlimb casting. Muscle properties in response to SM had higher levels of heritability than in control, such as soleus mass and soleus/BW. These data support the notion that mouse genetic background dictates the majority of the skeletal muscle response to SM.

A/J and CAST/EiJ strains were the most susceptible to SM while NOD/ShiLtJ and NZO/HILtJ mice were the most protected against SM-induced atrophy. It is interesting to note that both of these strains were bred to be used as diabetic models and both had similar protective responses. NZO/HILtJ mice are an obese type II diabetic model, and NOD/ShiLtJ mice are a non-obese, type I diabetic model. These strains are prone to insulin resistance and development of diabetes as they age^100,101^. Type I diabetes involves a lack of insulin availability which leads to downregulation of the mTORC1 pathway^102^. Similarly, PI3k/Akt activation is decreased in type II diabetes^103^. These actions would be expected to accelerate disuse-atrophy in the NOD/ShiLtJ and NZO/HILtJ mice. Unexpectedly, we did not observe increased muscle atrophy in these mouse strains. However, the blood sugar levels of the mice were not measured throughout the study, and thus, we were unable to verify whether any mice fully developed diabetes. It is likely that some of the NZO/HILtJ mice were in the prediabetic or early diabetic stage as male NZO/HILtJ mice develop hyperglycemia around 8–12 weeks of age^104^. 30–40% of NOD/HILtJ mice develop diabetes by 30 weeks^105^, reducing the likelihood of the NOD/HILtJ mice having impaired insulin signaling during the duration of the experiment and potentially explaining the lack of expected sensitivity to SM. The NZO/HILtJ mice, in particular, showed significant upregulation of several anabolic muscle GOs including sarcomere organization, muscle fiber development, and striated muscle contraction (Table 3), suggesting that this strain has intrinsic compensatory pathways against unloading of the muscle. Further, it is possible that the HLU model we used, which increased freedom to contract the legs, protected NOD/ShiLtJ and NZO/HILtJ mice from the effects of impaired insulin signaling.

There were some limitations to this study. Our sample sizes were smaller than expected due to the stress experienced by some mouse strains in HLU (129S1/SvImJ, A/J, and NOD/HILtJ) and pair housing (PWK/PhJ and WSB/EiJ). Only male mice were used in this study to reduce the number of animals needed. However, skeletal muscle disuse atrophy is reported to be more pronounced in females compared to male mice^106–108^. In the future, both males and females should be considered as genetic variation due to sex differences could also influence the skeletal muscle response to microgravity. Indeed, we have found this to be the case in bone response to disuse^109^. Since the strains used in this study are inbred, they do not fully reflect the genetic diversity seen in the DO mice. Discrepancies between muscle mass data and volume and area data can be attributed to differential responses between different muscles to SM. Since the micro-CT scanner resolution does not allow for the delineation between muscles such as the soleus, gastrocnemius, and tibialis anterior, individual changes in muscle CSA and volume could not be analyzed. Moreover, the absence of histological data prevented the ability to quantify changes in fiber type composition and how these changes could have varied by strain. Several murine studies support the notion that lower leg muscles undergo compositional shifts from Type I to Type II fibers in unloading^110–114^. It is important to note that differences in physical properties between Type I and Type II muscle fibers could impact muscle density and mass. These observations underscore the complexity of the skeletal muscle response to SM and highlight the need for more comprehensive studies to understand the underlying mechanisms behind the genetic impact on skeletal muscle response to SM. Exploring how SM affects DO mice will be necessary to better understand the role of genetic variation and discover causal genes regulating the skeletal muscle response to SM.

In summary, genetics influence the skeletal muscle response to SM in male mice. For long-term missions to the Moon and Mars, countermeasure strategies to protect skeletal muscle against loss of mass and strength should take genetic background into account and be individualized for each astronaut. Further, our results suggest that more research is needed to determine the role of the immune system in mediating unloading-induced skeletal muscle atrophy, since spaceflight causes immune dysfunction and could potentially exacerbate the skeletal muscle loss seen in astronauts. Future studies should use DO mice to better understand the specific genes and alleles that regulate the skeletal muscle response to unloading and identify protective genes that could assist the development of interventions to prevent unloading-induced muscle atrophy. Potential gene targets identified in this study include *Dusp8, Chrbn1*, and *Nus1*.

## Methods

### Animals

All animal procedures were completed with approval from the Virginia Commonwealth University Institutional Animal Care and Use Committee (Protocol # AD10001341). The protocols we used followed ethical regulations for animal testing and research. We used the eight founder strains of DO mice from the Jackson Laboratory (JAX, Bar Harbor, ME, USA: 129S1/SvImJ (stock #002448), A/J (stock #000646), C57BL/6 J (stock #000664), CAST/EiJ (stock #000928), NOD/ShiLtJ (stock #001976), NZO/HILtJ (stock #002105), PWK/PhJ (stock #003715), and WSB/EiJ (stock #01145). Sixteen male mice between four and fourteen weeks old from each of the eight strains were purchased from JAX). They were given one week to acclimate to the animal facility and another week to acclimate to the wire-bottom cage environment. At sixteen weeks, half the mice from each strain were placed into SM; the other eight were left as age-matched ground controls. Mice were pair-housed in standard rat cages with wire floors. Mice were fed Teklad LM-485 chow (Envigo) and water ad libitum and kept on a 12-h light and dark cycle. The mice in HLU were provided five pellets of chow at a time to prevent them from using their food as leverage to walk on while suspended. The duration of the study was three weeks, after which the mice were sacrificed, and skeletal muscle tissue was immediately isolated for analysis.

### Simulated microgravity protocol

HLU was used as the SM model for this experiment as it unloads the hindlimbs and mimics the cephalic fluid shift that occurs in true microgravity^115^. In addition, unlike hindlimb immobilization, HLU allows for uninhibited leg muscle contractions to occur during unloading, but no reaction forces are present to act against the muscles. The SM protocol was based off of a modified version of the Morey-Holton model^116^. Two cross bars were added to the top of each half of the rat cages. Under general anesthesia (1–3% v/v isoflurane), the tails were wiped down with ethanol, dried, and then fully wrapped with two pieces of surgical tape. The loose ends of the tape were attached to a swivel hook attached to the end of a string that was wrapped around the center of the cross bar. The string was wound up until the animals’ hindlimbs reached an elevation of 30°. This model prevents strain on the tails of the mice while maintaining a normal load on the forelimbs, reducing stress^117^. Since the mice were restrained due to HLU, they were unable to make contact with each other. Lab and veterinary staff inspected the condition of the mice daily. If strings were unwound from the bars, changing the angle of elevation, the mice were anaesthetized to reattach the strings properly and resuspend the mice. The body weights (BW) of the mice were measured every 7–10 days to monitor their health, but only the data from days 1 and 23 of SM were used for analysis. Mice were removed from the study, after consultation with the veterinary staff, if they lost more than 20% of their body weight or experienced other health issues.

### Muscle morphology

In vivo microCT scanning was performed to analyze changes in skeletal muscle morphology. CT has been shown to be a reliable method to assess appendicular skeletal muscle morphology^118^. On days 1 and 23 of the procedure, mice were anaesthetized with isoflurane (2.5%) and underwent in vivo microCT scanning (SkyScanner 1276, Bruker, Kontich, Belgium). The animals’ hindlimbs were extended until they were straight, and then they were taped down. Their snouts were placed in a nose-cone to maintain general anesthesia by isoflurane. The scans were taken with a resolution of 60μm, exposure time of 73 ms, a current of 200 µA, source voltage of 60 kV, and a 0.5 mm aluminum filter. Average scan time of the hindlimbs lasted 10–15 s and resulted in less than 50mGy of radiation exposure. The scans were reconstructed using NRecon as well as the GPUReconServer reconstruction engine. Reconstructions were processed and oriented using anatomical landmarks with Dataviewer. The region of interest spanned the entire length of the tibia. Skeletal muscle cross-sectional area and volume were analyzed using CTAn with manually segmented regions of interest. Bone and subcutaneous fat were excluded from the analysis based on attenuation. Total muscle cross-sectional area was analyzed and reported as the average total area of all muscles throughout the lower leg in mm^2^; total muscle volume was analyzed and reported as mm^3^.

### Muscle force testing

On day 23, the mice were placed under anesthesia with isoflurane (2.5%) and clippers were used to shave their hindlimbs. Muscle performance was measured in vivo using a muscle lever system (1300 A system, Aurora Scientific Inc., Aurora, CAN). The mice were laid onto a heated platform and had their snouts placed in a nose-cone to maintain anesthesia. The left leg was locked into place with a knee clamp and the foot was fixed onto a 10 N pedal force transducer at a 90° angle. Plantar flexion was induced through percutaneous electrical stimulation of the tibial nerve using two monopolar electrodes. Starting at 10 mA, the current was increased until reaching the force response plateaued at maximum strength to find the optimal isometric twitch torque. Twitch force (under 40 Hz 300 ms pulse) was measured three times with five second rest periods in between. Maximum twitch force was calculated using the Aurora 605 A: Dynamic Muscle Data Acquisition Analysis System. The three twitch force measurements were averaged into one value for analysis. Due to technical issues encountered during initial measurements of muscle strength, only data for C57BL/6J and A/J mice were used for analysis.

### RNA sequencing

The gastrocnemius was extracted from the animals and incubated in RNA Later (Thermo Fisher Scientific, Cleveland, Ohio, USA) in a 4 °C refrigerator overnight. The following day the RNA Later was removed, and the samples were stored in a −80 °C freezer. A 20–30 mg slice of gastrocnemius muscle was used for RNA extraction. Muscle sample total RNA was extracted with the RNeasy Fibrous Tissue Kit (Qiagen, Valencia, CA, USA) using the manufacturers’ instructions. The RNA samples were treated with DNase (Iscript gDNA Clear cDNA Synthesis Kit, Bio-Rad, Hercules, CA, USA) and tested for RNA Integrity (2100 Bioanalyzer, Agilent, Santa Clara, CA). Only samples with an RNA Integrity Number of at least 5.0 were used. Differentially expressed genes (DEGs) with a false discovery rate (FDR) below 0.05 were used for analysis. Six samples from each strain, three control and three SM, were sequenced at the VCU Genomics Core facility using a NextSeq2000 Sequencer (Illumina). The samples were treated with the Illumina Stranded mRNA Prep to purify and fragment mRNA. Then, cDNA was synthesized, and the 3’ ends were adenylated. The samples were then treated with a ligation kit before being amplified and sequenced.

### Western blot analysis

Protein isolation was completed using 20 mg of tissue from the quadriceps. The samples were homogenized in the buffer our lab used previously^37^. Quantification of protein concentration was done using a BCA kit (Thermo Fisher Scientific, Cleveland, Ohio, USA). 30 μg of total protein from each sample was placed into Mini-PROTEAN (R symbol) TGX (™) 4–20% gels (Bio-Rad) and subjected to SDS-PAGE. The gels were then electroblotted onto PVDF membranes (Bio-Rad). Western blot analysis was performed to assess total and phosphorylated p70S6K1 (70 kDa ribosomal protein S6 kinase) (T389, Cell Signaling Technology; Boston, MA) as well as 4EBP1 (4E-binding protein 1) (T37/46, Cell Signaling Technology). Primary antibodies were diluted at a 1:1000 ratio, and secondary antibodies were diluted 1:3000. Clarity Max (™) Western ECL Substrate (Bio-Rad) was used to detect the immune complexes. Protein quantification was completed using Image Lab software (version 6.1). To account for variation in protein loading, phosphorylated protein was normalized to total protein and the resulting ratio was used for statistical analysis. Blots can be seen in the Supplementary Information file.

### Statistical analysis

All statistical analyses were completed on Prism (version 9.0; GraphPad Software, La Jolla, CA). Data were analyzed using two-way ANOVAs with significance set at *p* < 0.05 to evaluate the main effects of mouse strain, SM, and interactions. Narrow-sense heritability was calculated using one-way ANOVA to evaluate the additive effect of genetics on muscle properties, as previously described^37,119^. Tukey’s multiple comparisons post-hoc test was used for data with repeated measures. Biojupies was used to perform statistical analyses on the data from RNA sequencing, including analyses for differentially expressed genes, gene ontologies, and pathway enrichment^120^. RNA sequencing analyses included comparing SM vs. control for each individual mouse strain, as well as combining all eight strains and analyzing SM vs. control.

### Supplementary information

Supplementary Information

Supplementary Data File 1

Supplementary Data File 2

## Data Availability

All data generated or analyzed during this study are included in this published article and its supplementary information files.
